# Observational cohort study of IP-10’s potential as a biomarker to aid in inflammation regulation within a clinical decision support protocol for patients with severe COVID-19

**DOI:** 10.1371/journal.pone.0245296

**Published:** 2021-01-12

**Authors:** Shaul Lev, Tamar Gottesman, Gal Sahaf Levin, Doron Lederfein, Evgeny Berkov, Dror Diker, Aliza Zaidman, Amir Nutman, Tahel Ilan Ber, Alon Angel, Lior Kellerman, Eran Barash, Roy Navon, Olga Boico, Yael Israeli, Michal Rosenberg, Amir Gelman, Roy Kalfon, Einav Simon, Noa Avni, Mary Hainrichson, Oren Zarchin, Tanya M. Gottlieb, Kfir Oved, Eran Eden, Boaz Tadmor

**Affiliations:** 1 Intensive Care Unit, Rabin Medical Center, Petah Tikva, Israel; 2 Sackler Faculty of Medicine, Tel Aviv University, Tel Aviv, Israel; 3 Department of Infectious Diseases and Infection Control Unit, Rabin Medical Center, Petah Tikva, Israel; 4 Rabin Medical Center, Petah Tikva, Israel; 5 Department of Internal Medicine, Rabin Medical Center, Petah Tikva, Israel; 6 National Institute for Infection Control and Antibiotic Resistance, Tel Aviv Medical Centre, Tel-Aviv, Israel; 7 MeMed, Haifa, Israel; Heidelberg University Hospital, GERMANY

## Abstract

**Background:**

Treatment of severely ill COVID-19 patients requires simultaneous management of oxygenation and inflammation without compromising viral clearance. While multiple tools are available to aid oxygenation, data supporting immune biomarkers for monitoring the host-pathogen interaction across disease stages and for titrating immunomodulatory therapy is lacking.

**Methods:**

In this single-center cohort study, we used an immunoassay platform that enables rapid and quantitative measurement of interferon γ-induced protein 10 (IP-10), a host protein involved in lung injury from virus-induced hyperinflammation. A dynamic clinical decision support protocol was followed to manage patients infected with severe acute respiratory syndrome coronavirus 2 and examine the potential utility of timely and serial measurements of IP-10 as tool in regulating inflammation.

**Results:**

Overall, 502 IP-10 measurements were performed on 52 patients between 7 April and 10 May 2020, with 12 patients admitted to the intensive care unit. IP-10 levels correlated with COVID-19 severity scores and admission to the intensive care unit. Among patients in the intensive care unit, the number of days with IP-10 levels exceeding 1,000 pg/mL was associated with mortality. Administration of corticosteroid immunomodulatory therapy decreased IP-10 levels significantly. Only two patients presented with subsequent IP-10 flare-ups exceeding 1,000 pg/mL and died of COVID-19-related complications.

**Conclusions:**

Serial and readily available IP-10 measurements potentially represent an actionable aid in managing inflammation in COVID-19 patients and therapeutic decision-making.

**Trial registration:**

Clinicaltrials.gov, NCT04389645, retrospectively registered on May 15, 2020.

## Introduction

In most severe acute respiratory syndrome coronavirus 2 (SARS-CoV-2)-positive patients, a localized, short-lasting immune response is sufficient to clear the virus from the lungs, following which the immune response recedes and the patient recovers [1]. In fewer than 15% of patients, a dysregulated immune response ensues, triggering a hyperinflammatory state [2,3] that can lead to acute lung injury, acute respiratory distress syndrome (ARDS), multiple organ failure, and mortality [4–7]. This exaggerated inflammatory response resembling secondary hemophagocytic lymphohistocytosis is characterized by increased interleukin (IL)6, IL-2, IL-7, granulocyte colony-stimulating factor, interferon γ-induced protein 10 (IP-10, also termed CXCL-10), monocyte chemoattractant protein 1, macrophage inflammatory protein 1 α, and tumor necrosis factor α [2]. Accumulating evidence points specifically to IP-10 as a marker of coronavirus disease 2019 (COVID-19) progression, with maintained high levels associated with mortality [8–15]. In various viral infection models, IP-10 levels are elevated [16–19] and implicated initially as promoting viral clearance [20], but also, as an effector of immune-mediated acute lung injury, suggesting it plays a key role in both the normal and dysregulated responses to SARS-CoV-2 infection [21–23]. Accordingly, IP-10 may represent a marker for COVID-19 disease progression and a target in preventing lung injury.

Treating severely ill COVID-19 patients involves simultaneous management of oxygenation and inflammation without compromising viral clearance. The challenge is further exacerbated by an evolving understanding of the complex host-pathogen interaction and by limited medical resources, leading to variations in how patients are managed across facilities and locations [24]. The present study was performed during the first COVID-19 wave in Israel, which peaked in April/May 2020. At that time, although evidence supported suppressing the immune system in severe COVID-19 patients to mitigate cytokine release [25], there was concern that over-suppression may reduce the immune system’s capacity to respond effectively to the virus and increase susceptibility to secondary infections [26]. Corticosteroids in general and methylprednisolone in particular are strong non-specific immunomodulatory agents, and their use in severe COVID-19 patients was, at that time, a topic of ongoing debate and clinical research [25–29]. Several tools are available for guiding therapeutic decisions on oxygenation but there are, still today, no well-established and actionable biomarkers for monitoring the host-pathogen interaction, the ensuing inflammatory response, and its modulation by corticosteroids. This unmet need is underscored by the recent consensus, based on the landmark RECOVERY study, that corticosteroids are indeed beneficial for critically ill patients with COVID-19 receiving oxygenation [30,31].

During the first COVID-19 wave, we developed a clinical decision support protocol aimed at achieving three predefined patient management goals: improving oxygen delivery, controlling inflammation, and promoting viral clearance. We hypothesized that real-time IP-10 measurements can support identification and continuous monitoring of patients with a dysregulated immune response and can potentially enable personalization of corticosteroid regimens to improve patient outcomes. This paper reports the longitudinal observation of serial IP-10 measurements as a potential tool in inflammation control.

## Material and methods

### Patient population

#### Study design

This study used the following cohorts: a prospective cohort of SARS-CoV-2-positive patients (n = 52) followed prospectively; (2) a retrospective cohort of adult patients with other respiratory viruses (n = 182) pooled from two prospective studies, CURIOSITY [16] (NCT01917461) and OBSERVER (NCT03011515); and a retrospective cohort of healthy adult patients (n = 98) pooled from the prospective studies CURIOSITY [16] (NCT01917461) and OBSERVER (NCT03011515).

#### COVID-19 patients

Fifty-two SARS-CoV-2-positive patients were followed prospectively at a COVID-19-dedicated facility, Rabin Medical Center, Hasharon, Petah Tikva, managed by Clalit Health Services, the second largest Health Management Organization worldwide. The inclusion criteria were a SARS-CoV-2 positive test result, hospital admission to the site between 7 April and 10 May 2020, and application by the attending physician of the clinical decision support protocol. This population is considered representative of SARS-CoV-2 patients admitted during the first COVID-19 wave in Israel. Hasharon institutional review board approval was obtained (Approval #0257-20-RMC) and informed consent was waived as biomarker measurements were performed on serum remnants from routine blood draws. The study was registered in ClinicalTrials.gov as NCT04389645. Acute respiratory distress syndrome and its severity were defined according to the Berlin definition [32].

#### Patients with other respiratory viruses

A retrospective analysis was performed on data from a cohort of adult patients (n = 182) pooled from two prospective studies, CURIOSITY [16] (NCT01917461) and OBSERVER (NCT03011515). The CURIOSITY study recruited 1,002 patients between August 2009 and November 2013 from Hillel-Yaffe and Bnai-Zion Medical Centers, Israel. The OBSERVER study recruited 583 patients between March 2017 and October 2018 from Rambam Health Care (Haifa), Carmel Medical Center (Haifa), and Rabin Medical Center (Beilinson Hospital), Petah Tikva, Israel. Patients were included if they met the inclusion criteria of the original study and had tested positive for at least one of the following viruses: human rhinovirus, respiratory syncytial virus, influenza A or B, or human coronavirus. In addition, patients were included only if their diagnosis had been adjudicated as a viral infection by an expert panel (i.e., no bacterial infection had been detected).

#### Healthy subjects

A retrospective analysis was performed on data from a cohort of adult patients (n = 98) pooled from two prospective studies, CURIOSITY [16] (NCT01917461) and OBSERVER (NCT03011515). Patients were included if they were afebrile with no apparent infectious disease.

#### Clinical decision support protocol used in treating COVID-19 patients during the first wave

**Fig 1:**
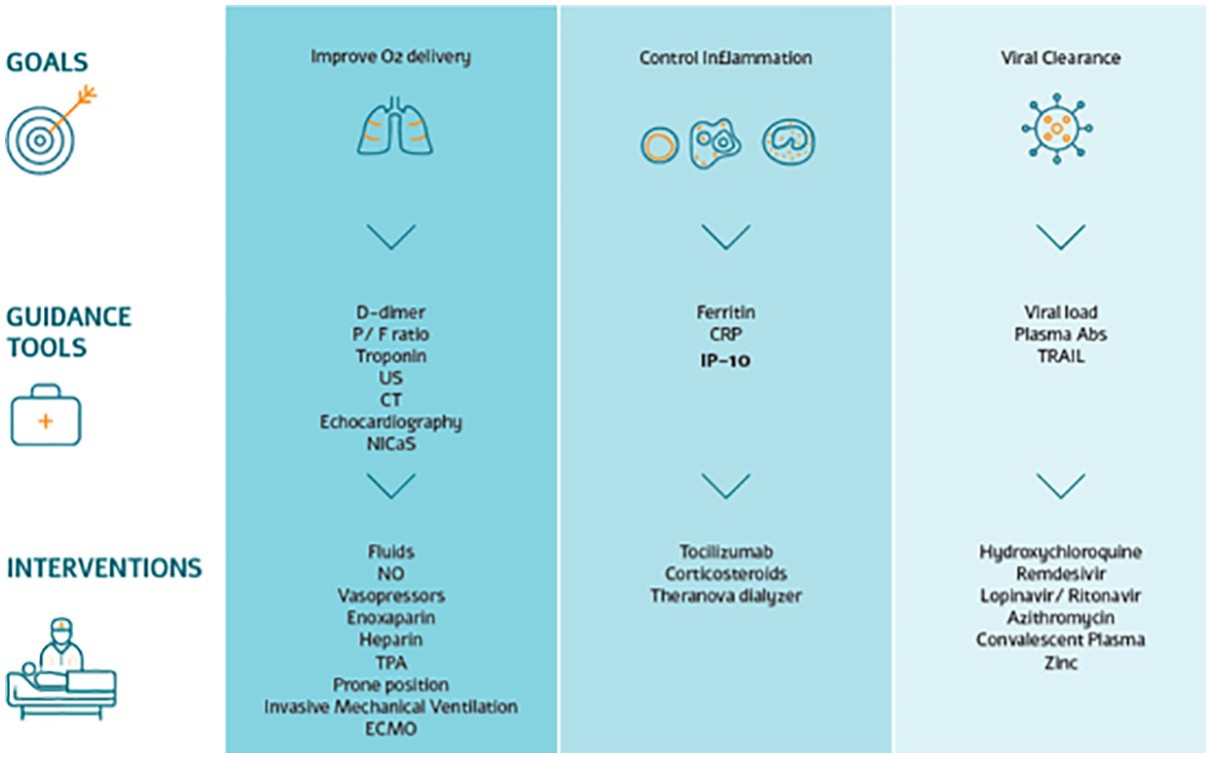
Overview of the dynamic clinical decision support protocol used in managing SARS-CoV-2 patients admitted to a COVID-19-dedicated medical center. Note that at the time the study was performed, multiple treatments and clinical decision protocols were being developed globally and employed despite the limited data to support improved outcomes and the research use only indications. P/F ratio, ratio of arterial oxygen partial pressure (P_a_O_2_) to fractional inspired oxygen (F_i_O_2_); US, ultrasound; CT, computed tomography; NICaS, non-invasive cardiac system; NO, nitric oxide; TPA, tissue plasminogen activator; ECMO, extracorporeal membrane oxygenation; CRP, C-reactive protein; IP-10, interferon γ-induced protein 10; Plasm Abs, plasma antibodies; TRAIL, tumor necrosis factor-related apoptosis-inducing ligand.

Fig 1 presents an overview of the clinical decision support protocol used to guide patient treatment during the first COVID-19 wave. Note that at the time the study was performed, multiple treatments and clinical decision protocols were being developed globally and employed despite the limited data to support improved outcomes and the research use only indications.

To manage oxygenation, the ratio of arterial oxygen partial pressure to fractional inspired oxygen was monitored twice daily and positive end-expiratory pressure titration was made based on cardiac output measurements when indicated. All patients received high-dose N-acetylcysteine and bromhexine inhalations to induce mucolysis and prevent sticky secretions and atelectasis. Intratracheally injected surfactant was an option as compassionate therapy for refractory hypoxemic ventilated patients.

Echocardiography and bedside Doppler ultrasound were ordered when pulmonary emboli or deep-vein thrombosis were clinically suspected and when D-dimer levels increased substantially. Troponin levels were measured daily and electrocardiogram measurements were performed routinely to detect QT prolongation.

All intensive care unit (ICU) patients received 40 mg of the anticoagulant enoxaparin sodium twice daily; the dose was adjusted up to 60 mg twice daily when D-dimer levels increased substantially. In case of thrombosis confirmed by imaging, full anticoagulation therapy was recommended. When minor bleeding occurred, subcutaneous heparin was administered.

All ICU patients considered hyperinflammatory were administered tocilizumab (800 mg), given in two 400-mg doses 12 h apart. A hyperinflammatory state was defined as C-reactive protein (CRP) levels >90 mg/L or ferritin levels >500 ng/dL. All hyperinflammatory ICU patients were administered methylprednisolone or the equivalent dosing of another corticosteroid; basic dosing was 1.5 mg/kg divided into three doses. Patients on hemodialysis who were hyperinflamed had access to cytokine-removal hemodialysis.

Antiviral therapy was based on combinations of hydroxychloroquine, azithromycin, zinc, and lopinavir/ritonavir at the discretion of the infectious disease consultant and was reviewed daily. Patients who did not show clinical improvement were given convalescent plasma in two doses as a compassionate therapy.

Compassionate remdesivir became available at the end of April 2020. The drug was given to only one patient for a total of 7 days (a 200-mg loading dose followed by a 100-mg maintenance dose from day 2 onwards) and was stopped because of elevated markers of liver dysfunction.

#### Laboratory procedures for COVID-19 patient cohort

Routine blood draws were collected serially from the 52 patients into serum separating tubes. Serum was separated within 2 h of collection and measured on the MeMed Key^™^ (MeMed, Haifa, Israel) immunoassay platform that provides measurements of three immune-related proteins (IP-10, CRP, and tumor necrosis factor-related apoptosis-inducing ligand [TRAIL]), within 15 min using MeMed BV^™^ testing cartridges. Any unused serum was frozen at −20°C for up to 1 week and then transferred to storage at −80°C.

Frozen serum was used to determine retrospectively blood concentrations of IL-6. Measurements were performed using Quantikine human IL-6 enzyme-linked immunosorbent assay kits (R&D Systems, Minneapolis, MN, USA). Because IL-6 concentrations were high, each serum sample was diluted 1:4 and 1:40 with a calibrator diluent supplied in the kits. Results were averaged when the difference in concentration between the two dilutions was <20%; otherwise, the dilution in the linear range of the calibration curve was taken. Measurements were performed using a Sunrise absorbance microplate reader and the Magellan program (Tecan Life Sciences, Männedorf, Switzerland). SARS-CoV-2 positivity was determined by reverse transcription polymerase chain reaction (RT-PCR; Seegene Inc., Seoul, Korea).

#### Statistical analysis

Continuous variables are reported as the median and interquartile range. Group comparisons were performed using the Mann-Whitney U test (for continuous values) and Fisher’s exact test (for discrete values). Steroid doses were converted to methylprednisolone-equivalent doses using MDCalc’s Steroid Conversion Calculator [33]. The analysis was performed in Python 3.7.6.

## Results

### COVID-19 patient population

Overall, 502 serial blood draws were taken from 52 SARS-CoV-2 patients that were admitted to the COVID-19-dedicated facility, Rabin Medical Center, Hasharon, Petah Tikva, managed by Clalit Health Services between 7 April and 10 May 2020. Twelve of the patients were admitted to the ICU. The median age of the 52 patients was 69 years (range: 35.2–95.1 years), two thirds (36) were male, almost one quarter (12) were ventilated, and four died. There was low in-ICU mortality (one death) and no non-pulmonary organ failure. Three of the 12 ICU patients died within 90 days of admission. The most common comorbidities in the cohort were hypertension (24 patients) and diabetes (22 patients; Table 1).

**Table 1:** Characteristics of COVID-19 patients.

	All	Non-ICU	ICU	*p*-value
	(n = 52)	(n = 40)	(n = 12)	
**General**				
**Median age, years (interquartile range)**	68.6 (16.7)	66.4 (20.0)	70.2 (13.2)	0.313
**Min, Max age (years)**	35.2, 95.1	35.2, 95.1	46.1, 84.1	
**Female**	16	15	1	0.078
**Smoking status**				
**Smoker**	3	3	0	1
**Past smoker**	3	3	0	1
**Hospitalization details**				
**Death**	4	1	3	0.034
**Discharge**	51	39	12	1
**Highest temperature (°C)**	37.4 (1.2)	37.3 (0.9)	38.3 (0.9)	<0.001
**Duration of hospital stay (days)**	11.0 (13.5)	9.0 (7.5)	26.5 (12.2)	<0.001
**Time between PCR-positive and MeMed BV test (days)**	4.5 (6.0)	3.0 (5.0)	8.0 (8.8)	0.003
**Ventilated**	12	2	10	<0.001
**Time between symptom onset and BV test (days)**	7.0 (10.2)	6.0 (10.0)	14.0 (7.0)	0.063
**Time between symptom onset and ICU admission (days)**	8.0 (4.0)		8.0 (4.0)	
**Time between symptom onset and PCR-positive test (days)**	2.5 (5.0)	2.0 (4.5)	4.0 (5.2)	0.343
**Time on ventilator (days)**	0.0 (0.0)	0.0 (0.0)	15.0 (11.0)	<0.001
**Time between PCR-positive test and ICU admission (days)**	4.5 (2.2)		4.5 (2.2)	
**Time between PCR-positive test and PCR-negative test (days)**	19.0 (17.2)	8.5 (10.0)	26.5 (10.0)	0.015
**Co-infection**				
**Bacterial infection**	10	2	8	<0.001
**Co-infection**	10	2	8	<0.001
**Fungal infection**	4	0	4	0.002
**Other medical conditions**				
**Diabetes**	22	16	6	0.74
**Cardiovascular disease**	9	7	2	1
**Cerebrovascular disease**	3	3	0	1
**Chronic lung disease**	7	6	1	1
**Chronic renal disease**	6	4	2	0.612
**Dyslipidemia**	9	6	3	0.415
**Hypertension**	24	18	6	1
**Hypothyroidism**	6	2	4	0.021
**Malignancy**	5	2	3	0.074
**Obesity (body mass index > 30 kg/m**^**2**^**)**	9	8	1	0.666
**Immunodeficiency**	1	1	0	1
**Symptoms**				
**Sore throat**	1	1	0	1
**Vomiting**	2	1	1	0.412
**Urinary complaints**	1	1	0	1
**Sputum production**	1	1	0	1
**Weakness**	12	11	1	0.253
**Rhinorrhea**	1	1	0	1
**Lethargy**	2	2	0	1
**Agitation**	1	1	0	1
**Chest pain**	2	2	0	1
**Confusion**	2	2	0	1
**Chills**	1	0	1	0.231
**Diarrhea**	6	6	0	0.316
**Dyspnea**	23	17	6	0.746
**Fatigue**	3	2	1	0.553
**Fever**	29	18	11	0.007
**Headache**	5	4	1	1
**Loss of taste and/or smell**	5	5	0	0.578
**Myalgia**	4	4	0	0.562
**Nausea**	3	3	0	1
**Cough**	26	17	9	0.097
**Serum marker levels**				
**Aspartate transaminase (IU/L) max**	37.0 (38.8)	28.4 (25.5)	86.0 (91.2)	0
**Alanine aminotransferase (IU/L) max**	34.2 (70.1)	28.6 (27.0)	120.1 (147.1)	0
**Albumin (g/dL) max**	3.8 (0.5)	3.8 (0.5)	3.6 (0.3)	0.113
**International normalized ratio of prothrombin time max**	1.1 (0.2)	1.1 (0.2)	1.2 (0.2)	0.037
**Glucose (mg/dL) max**	153.5 (129.6)	126.4 (85.1)	247.0 (76.9)	<0.001
**Lactate dehydrogenase (IU/L) max**	610.0 (347.5)	500.0 (244.5)	912.5 (263.8)	<0.001
**Absolute lymphocytes (K/μL) max**	1.3 (0.8)	1.2 (0.6)	2.0 (3.9)	0.03
**Absolute lymphocytes (K/μL) min**	0.8 (0.7)	0.8 (0.7)	0.4 (0.4)	0.003
**Absolute neutrophils (K/μL) max**	5.6 (5.6)	5.0 (3.0)	13.8 (6.2)	<0.001
**Absolute neutrophils (K/μL) min**	3.1 (1.9)	3.1 (2.4)	3.0 (1.1)	0.214
**Platelets (10**^**3**^**) max**	375.0 (193.8)	319.0 (219.2)	408.5 (71.5)	0.062
**Platelets (10**^**3**^**) min**	186.5 (111.8)	228.0 (123.5)	139.5 (49.8)	0.001
**Ratio of arterial oxygen partial pressure to fractional inspired oxygen max**	284.0 (163.5)	166.0 (0.0)	313.5 (153.0)	<0.001
**Ratio of arterial oxygen partial pressure to fractional inspired oxygen min**	83.0 (56.5)	141.0 (0.0)	80.5 (42.0)	<0.001
**Total protein (g/dL) max**	7.0 (0.7)	7.0 (0.6)	7.0 (0.8)	0.136
**Troponin (ng/mL) max**	19.5 (44.5)	11.5 (18.5)	69.5 (55.5)	<0.001
**Urea (mg/dL) max**	46.3 (40.6)	37.2 (27.5)	105.0 (66.9)	<0.001
**Ferritin (ng/dL) max**	595.0 (1022.8)	462.0 (459.2)	1691.5 (916.8)	<0.001
**Ferritin (ng/dL) min**	283.0 (365.4)	277.2 (421.7)	330.2 (216.8)	0.368
**D-Dimer (ng/mL) max**	1319.0 (4050.0)	1140.0 (1377.5)	7235.5 (18598.2)	<0.001
**Creatinine (mg/dL) max**	1.0 (0.4)	0.9 (0.4)	1.0 (0.5)	0.086
**Total bilirubin (mg/dL) max**	0.5 (0.5)	0.5 (0.4)	0.9 (0.5)	0.001
**C-reactive protein (mg/L) max**	97.9 (122.8)	95.0 (92.1)	158.4 (172.6)	0.04
**C-reactive protein (mg/L) min**	19.6 (56.2)	29.9 (67.4)	2.2 (3.8)	<0.001
**C-reactive protein (mg/L) median**	70.9 (85.4)	77.8 (70.1)	36.0 (166.8)	0.5
**Scores**				
**COVID-19 severity**	26	14	12	<0.001
**Quick sequential organ failure assessment (on admission)**	0.0 (1.0)	0.0 (1.0)	1.0 (1.2)	0.003
**Treatments**				
**Solumedrol**	12 (23.0%)	1 (2.0%)	11 (92.0%)	<0.001
**Hydrocortisone**	10 (19.0%)	4 (10.0%)	6 (50.0%)	0.006
**Systemic steroids (intravenous or by mouth)**	22 (42.0%)	10 (25.0%)	12 (100.0%)	<0.001
**Renal replacement therapy**	2 (4.0%)	1 (2.0%)	1 (8.0%)	0.412
**Remdesivir**	1 (2.0%)	0 (0.0%)	1 (8.0%)	0.231
**Prednisone**	6 (12.0%)	6 (15.0%)	0 (0.0%)	0.316
**Nitric oxide**	5 (10.0%)	0 (0.0%)	5 (42.0%)	<0.001
**Extracorporeal membrane oxygenation**	1 (2.0%)	0 (0.0%)	1 (8.0%)	0.231
**Vasopressors**	11 (21.0%)	2 (5.0%)	9 (75.0%)	<0.001
**Convalescent plasma**	7 (13.0%)	3 (8.0%)	4 (33.0%)	0.041
**Azithromycin**	35 (67.0%)	24 (60.0%)	11 (92.0%)	0.076
**Antiviral lopinavir/ritonavir**	7 (13.0%)	1 (2.0%)	6 (50.0%)	<0.001
**Antiviral hydroxychloroquine plus azithromycin**	33 (63.0%)	22 (55.0%)	11 (92.0%)	0.037
**Antiviral hydroxychloroquine**	36 (69.0%)	24 (60.0%)	12 (100.0%)	0.01
**Antibiotics**	30 (58.0%)	19 (48.0%)	11 (92.0%)	0.008
**Tocilizumab**	13 (25.0%)	3 (8.0%)	10 (83.0%)	<0.001
**Days under systemic steroids**	0.0 (6.2)	0.0 (0.2)	7.5 (7.5)	<0.001

* COVID-19 severity score based on NIH treatment guidelines; COVID-19 Treatment Guidelines Panel. Coronavirus Disease 2019 (COVID-19) Treatment Guidelines. National Institutes of Health. Available at https://www.covid19treatmentguidelines.nih.gov/. Accessed August 2020.

Median values are followed by the interquartile range in parentheses. ICU, intensive care unit; PCR, polymerase chain reaction.

Among the 12 ICU patients, 10 required invasive mechanical ventilation and exhibited a pulmonary status compatible with severe acute respiratory distress syndrome (Table 2). Of these, seven were weaned during their ICU stay and transferred to a rehabilitation center, one was transferred to a respiratory rehabilitation center (Patient 5), and two (Patients 3 and 9) were transferred to the internal medicine department, where Patient 3 died. Eight patients developed secondary bacterial infections (Table 2).

**Table 2:** Status of the 12 patients admitted to the intensive care unit (ICU).

		Patient	1	2	3	4	5	6	7	8	9	11	18	38
ICU patient status		Age (years)	48	69	76	63	61	46	71	76	68	76	73	84
		Sex (M/F)	M	M	M	M	M	M	M	M	M	F	M	M
		Hypertension	no	no	**yes**	no	**yes**	no	no	no	**yes**	**yes**	**yes**	**yes**
		Diabetes	no	no	**yes**	no	**yes**	**yes**	no	no	**yes**	**yes**	no	**yes**
		Days in ICU	21	27	17	16	19	18	38	35	14	21	3	4
		Secondary bacterial coinfection	no	**yes**	**yes**	**yes**	no	**yes**	**yes**	**yes**	**yes**	**yes**	no	no
		Mortality	no	no	**yes**	no	no	no	no	**yes**	no	**yes**	no	no
Improve oxygen delivery	Guidance tools	Echocardiography	**yes**	no	**yes**	no	no	**yes**	**yes**	**yes**	no	**yes**	no	no
		D-Dimer > 10,000 ng/mL	**yes**	no	**yes**	no	no	no	**yes**	**yes**	**yes**	**yes**	no	no
		Ultrasound/computed tomography	no	no	no	no	no	no	**yes**	no	no	no	**yes**	no
		Troponin > 14 ng/mL	**yes**	**yes**	**yes**	**yes**	**yes**	**yes**	**yes**	**yes**	**yes**	**yes**	**yes**	**yes**
	Intervention	Nitric oxide	**yes**	no	**yes**	no	no	**yes**	no	**yes**	no	**yes**	no	no
		Prone position	**yes**	**yes**	**yes**	no	no	**yes**	**yes**	**yes**	no	**yes**	no	no
		Invasive mechanical ventilation	**yes**	**yes**	**yes**	**yes**	**yes**	**yes**	**yes**	**yes**	**yes**	**yes**	no	no
		Fluids	**yes**	**yes**	no	**yes**	**yes**	**yes**	**yes**	**yes**	**yes**	**yes**	**yes**	**yes**
		Vasopressor therapy	no	**yes**	**yes**	**yes**	**yes**	**yes**	**yes**	**yes**	**yes**	**yes**	no	no
		Extracorporeal membrane oxygenation	**yes**	no	no	no	no	no	no	no	no	no	no	no
		Enoxaparin	**yes**	**yes**	**yes**	**yes**	**yes**	**yes**	**yes**	**yes**	**yes**	**yes**	**yes**	**yes**
		Heparin	**yes**	**yes**	no	**yes**	no	**yes**	**yes**	**yes**	**yes**	**yes**	no	no
		Tissue plasminogen activator	no	no	**yes**	no	no	no	no	no	no	no	no	no
Control inflammation	Guidance tools	Ferritin > 500 mg/dL*	**yes**	**yes**	**yes**	**yes**	**yes**	**yes**	**yes**	**yes**	**yes**	**yes**	**yes**	**yes**
		C-reactive protein > 90 mg/L*	no	**yes**	**yes**	no	no	no	**yes**	**yes**	**yes**	**yes**	**yes**	**yes**
		Interferon γ-induced protein 10 > 1,000 ng/mL*	no	**yes**	no	**yes**	**yes**	no	no	**yes**	**yes**	**yes**	**yes**	**yes**
	Intervention	Tocilizumab	no	**yes**	**yes**	**yes**	**yes**	**yes**	**yes**	**yes**	**yes**	**yes**	no	**yes**
		Corticosteroids	**yes**	**yes**	**yes**	**yes**	**yes**	**yes**	**yes**	**yes**	**yes**	**yes**	**yes**	**yes**
Viral clearance	Guidance tools	Polymerase chain reaction (viral load)**	**yes**	**yes**	no	no	**yes**	**yes**	**yes**	**yes**	**yes**	no	**yes**	no
		Tumor necrosis factor-related apoptosis-inducing ligand <25pg/mL*	no	**yes**	**yes**	**yes**	no	no	no	**yes**	no	**yes**	no	**yes**
	Intervention	Zinc	no	no	**yes**	no	no	**yes**	**yes**	no	**yes**	no	no	no
		Lopinavir/ritonavir	no	**yes**	no	**yes**	**yes**	no	no	**yes**	no	**yes**	**yes**	no
		Remdesivir	no	no	no	no	no	no	no	no	no	**yes**	no	no
		Hydroxychloroquine	**yes**	**yes**	**yes**	**yes**	**yes**	**yes**	**yes**	**yes**	**yes**	**yes**	**yes**	**yes**
		Azithromycin	no	**yes**	**yes**	**yes**	**yes**	**yes**	**yes**	**yes**	**yes**	**yes**	**yes**	**yes**
		Convalescent plasma	no	**yes**	no	no	no	no	no	**yes**	no	**yes**	no	**yes**

*At least one measurement above/below the indicated level was required.

**Two negative SARS-CoV-2 polymerase chain reaction tests were required to confirm viral clearance.

### Clinical decision support protocol used during the first COVID-19 wave

A dynamic clinical decision support protocol was used to manage the SARS-CoV-2 patients admitted to the COVID-19-dedicated medical center (Fig 1). The protocol was based on an evolving understanding of the complex host-pathogen interaction during disease progression, and its objective was to improve oxygen delivery and control inflammation without compromising viral clearance. Note that at the time the study was performed, multiple treatments and clinical decision protocols were being developed globally and employed despite the limited data to support improved outcomes and the research use only indications. Observations relating to improving oxygen delivery are described in the supporting material. Applying the criterion of two negative SARS-CoV-2 PCR tests as confirmation of viral clearance, eight of the 12 ICU patients were clear of the virus within the study period (Table 2).

### IP-10 levels indicate hyperinflammation and are associated with mortality

**Fig 2:**
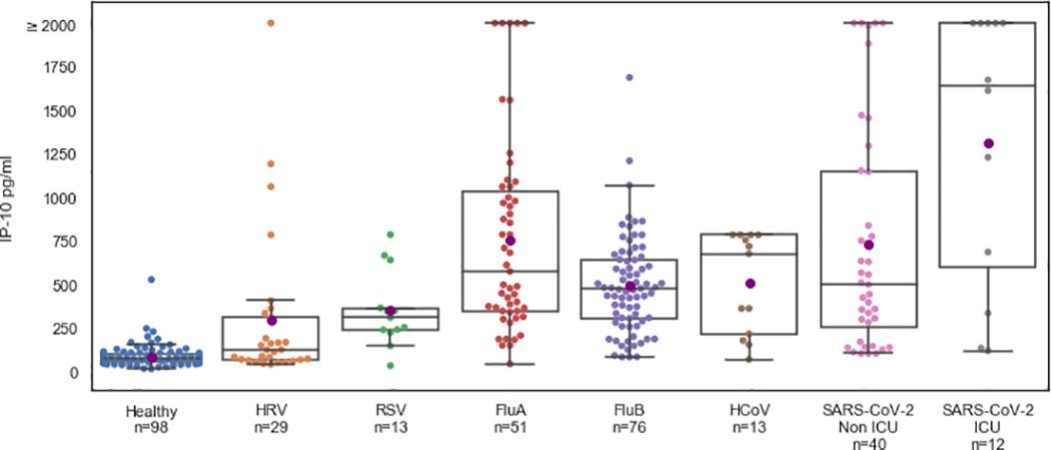
IP-10 levels are significantly higher in viral infections associated with pulmonary pathology. IP-10 levels in the internal medicine ward (non-ICU, n = 40) and ICU (n = 12) COVID-19 patients and in healthy subjects (n = 98) and virally infected patients (n = 182) with polymerase chain reaction-confirmed viral infections such as human rhinovirus (HRV), respiratory syncytial virus (RSV), influenza (Flu), and human coronavirus (HCoV). Each circle represents a patient. The black line denotes the group median and the purple circle corresponds to group mean. The box indicates patients with values between the 25 and 75 percentiles. The whiskers indicate patients with values between the 2.5 and the 97.5 percentiles. For the COVID-19 patients, IP-10 was measured using MeMed Key^™^ at various time points and the first IP-10 measurement is shown.

To compare IP-10 levels in SARS-CoV-2 patients, patients with other viral infections, and healthy subjects, we performed a retrospective multi-cohort analysis that included patients from previous studies (Fig 2). IP-10 levels were higher in patients with viral infections than in healthy subjects, 599.1 pg/ml (532.0–666.2) versus 82.9 pg/ml (70.8–95.1), respectively (*p* < 0.001). Moreover, IP-10 levels were higher when the viral infection was associated with a pulmonary pathology (e.g., respiratory syncytial virus, influenza A, influenza B, human coronavirus, and SARS-Cov-2) than when the infection had a non-pulmonary pathology (e.g., human rhinovirus; pairwise comparison, *p* < 0.02). Among the SARS-CoV-2-positive patients, IP-10 levels were much higher in ICU patients than in non-ICU patients, 1314.6 (870.6–1758.5) versus 732.0 (531.8–932.1), respectively (*p* < 0.019).

**Fig 3:**
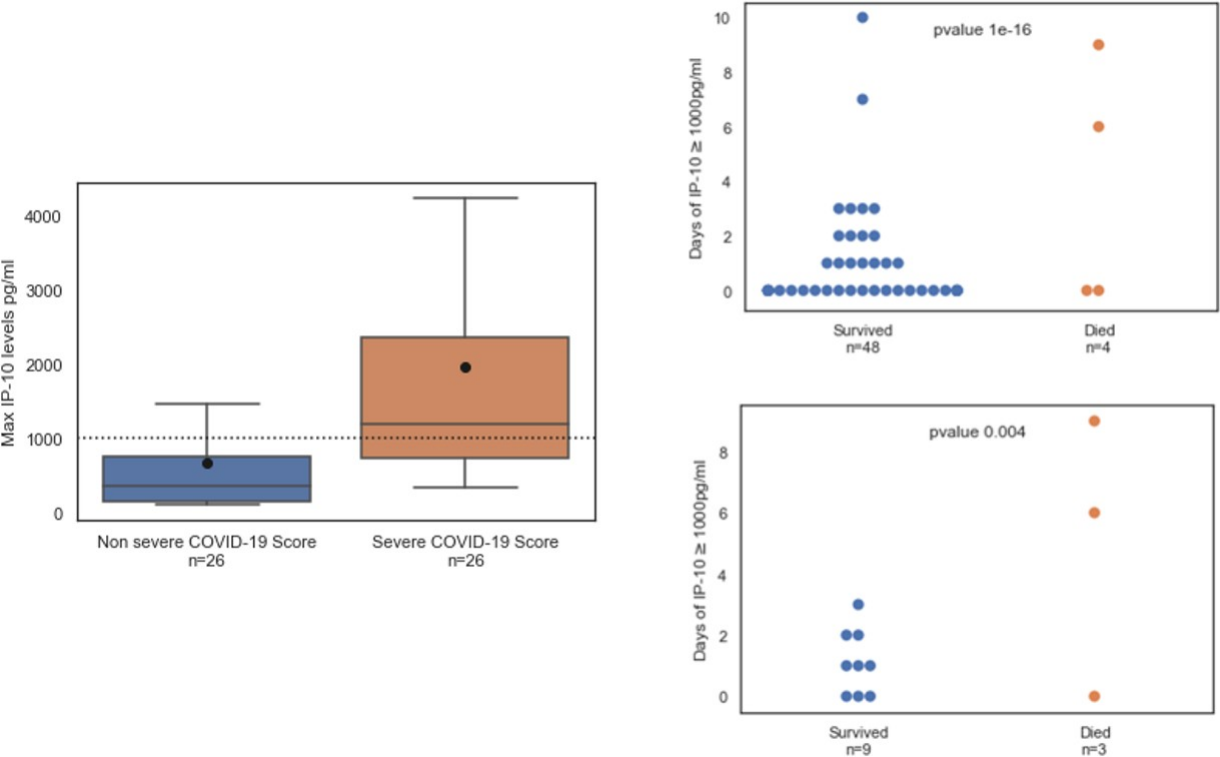
(Left panel) IP-10 levels correlate with COVID-19 severity. Maximal IP-10 levels in patients classified as non severe or severe according to the NIH COVID-19 severity score (https://www.covid19treatmentguidelines.nih.gov/. Accessed August 2020). The black line denotes the group median and the circle corresponds to group mean. The box indicates patients with values between the 25 and 75 percentiles. The whiskers indicate patients with values between the 2.5 and the 97.5 percentiles. (Right panel) Mortality correlates with the number of days when IP-10 levels exceeded 1,000 pg/mL in SARS-CoV-2-positive patients (n = 52, upper panel) and among the subset of intensive care unit (ICU) patients (n = 12, lower panel). IP-10 was measured at various time points during hospitalization. At least one IP-10 measurement ≥ 1,000 pg/mL in a given day was sufficient to classify the day as exceeding 1,000 pg/mL. Each circle represents a patient; blue dots represent patients who survived and red dots represent patients who died. The cause of death of the ICU patient with a low number of days of IP-10 > 1,000 pg/mL was candidemia and of the non-ICU patient was metastatic breast cancer.

Focusing on the prospective cohort of 52 SARS-CoV-2-positive patients, we found that IP-10 levels were significantly higher in patients classified as severe according to the NIH COVID-19 severity score (n = 26; https://www.covid19treatmentguidelines.nih.gov/. Accessed August 2020) versus the non severe patients (n = 26; Fig 3, upper panel). Accordingly, we found that IP-10 levels >1,000 pg/mL (threshold indicated by a dotted line in Fig 3A) correlated with an increased COVID-19 severity score (*p* < 0.01) and ICU admission (*p* < 0.05; S1 Table). Furthermore, in COVID-19 patients (n = 52) and the sub-group of ICU patients (n = 12), the number of days with IP-10 levels exceeding 1,000 pg/ml was associated with mortality (Fig 3, lower panel). In contrast, the number of days with CRP >90 mg/L or ferritin >500 ng/dL was not associated with mortality (S1 Fig). Among the 12 ICU patients, 11 were considered hyperinflammatory and 10 received tocilizumab and methylprednisolone; Patient 18 was admitted to the ICU after pneumothorax surgery and was not administered tocilizumab (Table 2). Overall, three ICU patients died; Patient 3 died of candidemia. In the other two ICU patients who died, IP-10 levels exceeded 1,000 pg/mL for an average of 7.5 days (standard deviation: 2.1 days). In the nine surviving patients, IP-10 levels exceeded 1,000 pg/mL for an average of 1 day (standard deviation: 1.1 day; *p* = 0.04).

**Fig 4:**
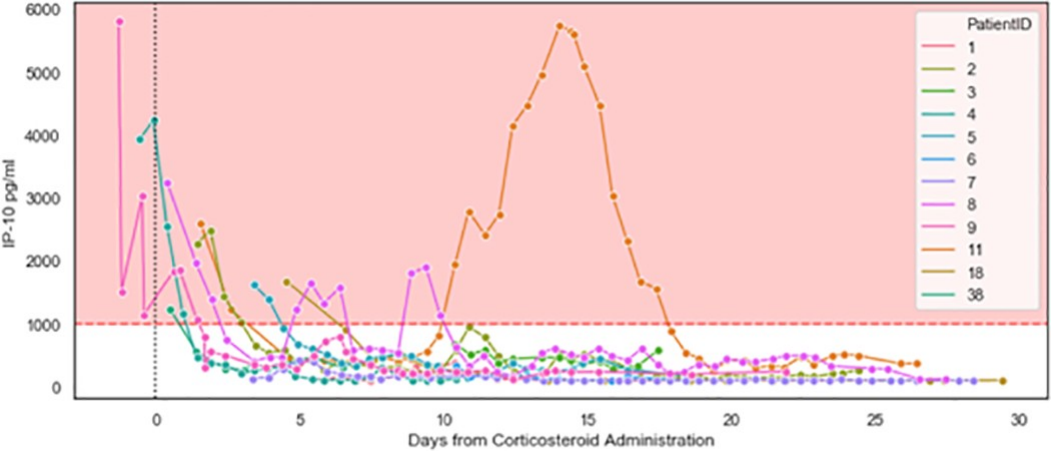
Initiation of corticosteroid therapy resulted in a decrease in IP-10 levels (ICU patients, n = 12). IP-10 was measured at the indicated time points during ICU stays. Day 0 indicates initiation of corticosteroid therapy. The pink area indicates IP-10 levels exceeding 1,000 pg/mL. Patients 2, 8, and 11 exhibited subsequent surges in IP-10 levels and are detailed in Fig 5. Of note, at study initiation, some patients were studied who were already hospitalized in the wards and ICU. Corticosteroid treatment was not initiated prior to hospitalization. From the point of patient selection, every measurement taken is included in the graph.

**Fig 5:**
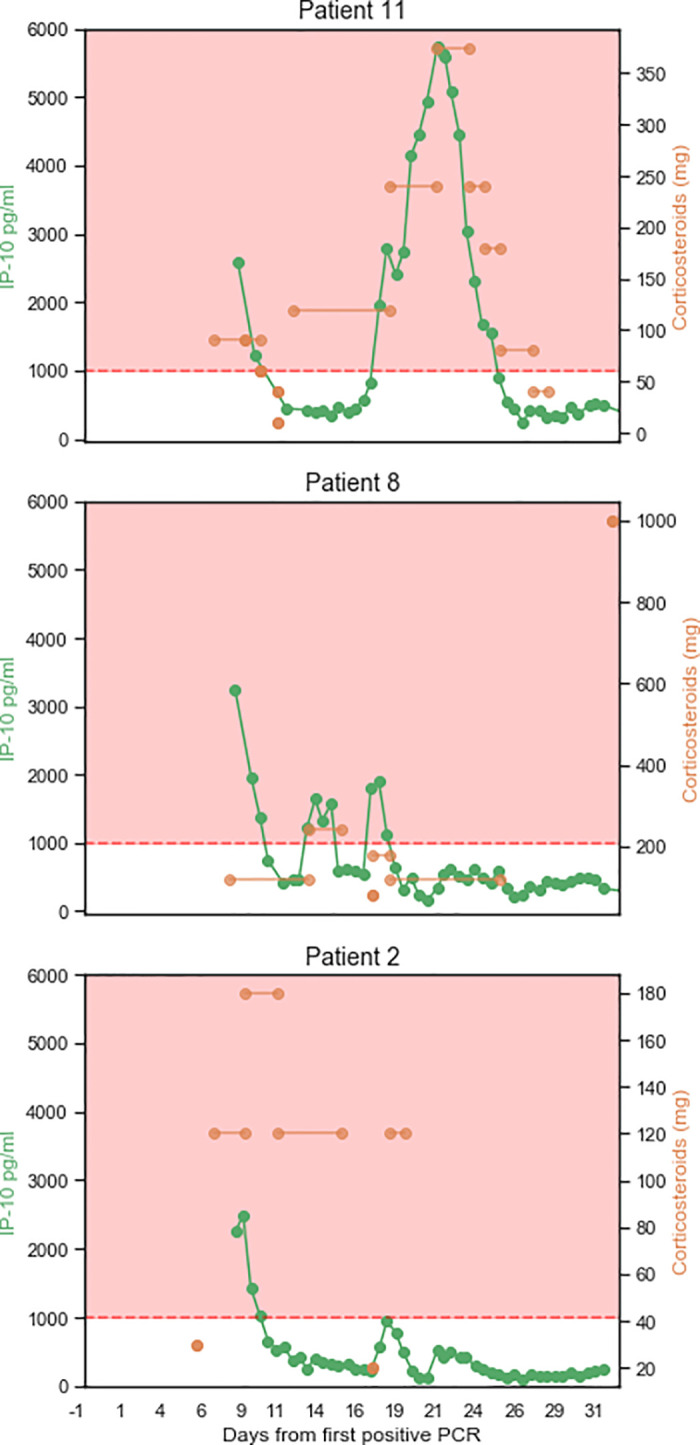
IP-10 levels reflect personalized corticosteroid dosing. Three patients exhibited a relapse wherein their IP-10 levels surged to exceed 1,000 pg/mL after an initial treatment with corticosteroids. The right Y axis shows the normalized levels of corticosteroids administered (the hydrocortisone dose was converted to a methylprednisolone dose). The left Y axis shows the levels of IP-10 measured at the indicated time points. The X axis shows the days from the first positive SARS-CoV-2 polymerase chain reaction test. Lines connecting the doses indicate that the dose was given in each of the intervening days. The pink area indicates IP-10 levels exceeding 1,000 pg/mL. Patient 2 survived; Patients 8 and 11 died.

All ICU patients exhibited reduced IP-10 within 3 days of their first corticosteroid treatment administered in the ICU (Fig 4); this was not observed for CRP and ferritin (S2 Fig). Notably, focusing on the 11 patients with sequential biomarker measurements within 48 hours before and after corticosteroid treatment, the average rate of IP-10 reduction was 597.1pg/ml*day (224.7–969.4 95%CI), as compared to an average rate of CRP reduction of 17.2mg/ml*day (-2.5–36.9 95%CI), the latter not significantly different from zero. For those patients that received additional corticosteroid dosing, a decrease in IP-10 levels was observed (Fig 5), although Patients 8 and 11 experienced multiple days of highly elevated IP-10 levels and died. Retrospective examination of IL-6 levels in the ICU patients identified a weak correlation with IP-10 levels (Pearson’s correlation coefficient: 0.26) and no association with mortality for number of days with IL-6 levels exceeding 80 pg/mL (*p* = 0.1) or 100 pg/mL (*p* = 0.1; S3 Fig).

### TRAIL levels correlate with viral positivity and indicate disease severity

**Fig 6:**
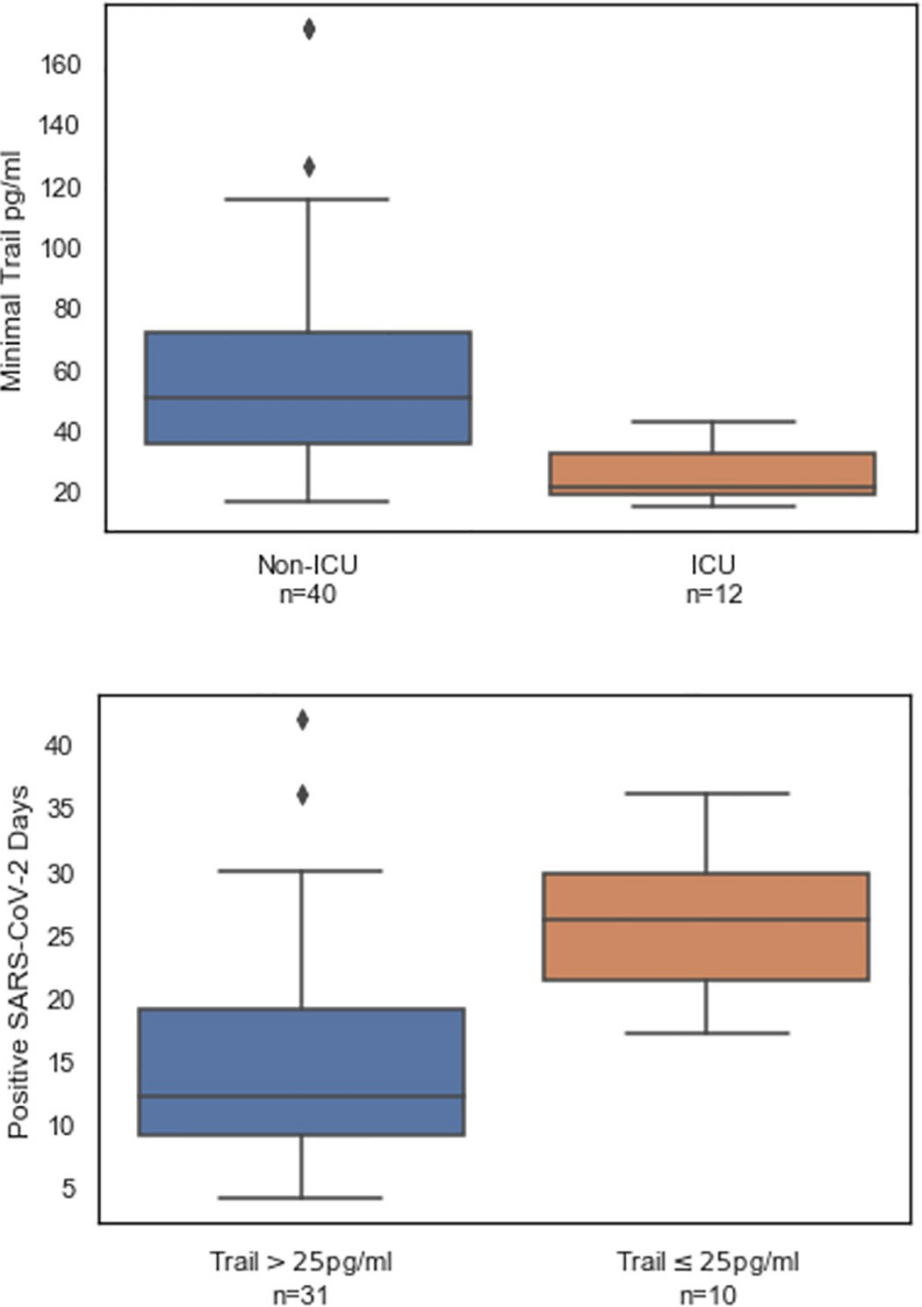
TRAIL levels correlate with ICU admission (upper panel) and viral positivity (lower panel). TRAIL was measured at multiple time points during hospitalization. At least one TRAIL measurement exceeding 25 pg/mL in a given day was sufficient to classify the day as exceeding 25 pg/mL. The bottom, middle and top box lines indicate the 25%, 50%, and 75% percentiles, respectively. The whiskers indicate the 25% and 75% quartile plus 1.5 times the interquartile range.

TRAIL is involved in the innate immune response to infection and has been shown to correlate with disease severity [34–36]. To test the hypothesis that low TRAIL levels correlate with viral persistence and patient deterioration, we analyzed TRAIL measurements and the time span of viral positivity. Patients admitted to ICU had lower average minimal TRAIL levels (25.7, 95%CI 20.1–31.3) compared to patients not admitted to ICU (60.2, 95%CI 48.6–71.8; Fig 6 upper panel). Of the 52 patients, 41 had at least two SARS-CoV-2-positive PCR results, with an average viral positivity time of 17.9 (15.0–20.9 95%CI). Patients with at least one measurement of TRAIL levels below 25 pg/mL exhibited an average time of viral positivity of 26.1 (21.9–30.3 95%CI) versus 15.3 days (12.1–18.5 95%CI) for the patients for whom TRAIL did not fall below this threshold (Fig 6, lower panel).

## Discussion

This is the first study to examine the potential utility of serial IP-10 measurements within a clinical decision support protocol aimed at improving oxygen delivery and controlling inflammation without compromising viral clearance or increasing susceptibility to secondary infections. The study was prompted by the need for additional, specific tools to monitor and assess the inflammatory status in COVID-19 patients. There is mounting evidence that aberrantly high IP-10 levels are directly involved in the acute lung injury observed in COVID-19 patients and that abnormally elevated IP-10 levels contribute to mortality. In line with this emerging understanding of the complex and multifactorial pathophysiology of COVID-19, we found that, unlike the levels of CRP and IL-6, IP-10 levels are associated with disease severity and reflect the patient’s response to corticosteroids. Specifically, we observed that relapsing surges of abnormally elevated IP-10 levels were characteristic of the patients that subsequently died, possibly because of more extensive lung damage. These findings suggest that longitudinal and readily available IP-10 measurements may assist in personalizing immunomodulatory treatment regimens for COVID-19 patients, leading to better patient outcomes.

The clinical decision support protocol described in this manuscript was developed during the first COVID-19 wave in parallel with the Yale New Haven Health treatment algorithm (version: June 22) [37] and the Massachusetts General Hospital COVID-19 treatment guidance (version: June 24) [38]. Similarly to our protocol, the June 22 Yale New Haven Health algorithm recommends interventions to address oxygenation, inflammation, and viral clearance. Notably, the Yale algorithm for hospitalized adult patients with severe disease recommends that a cytokine test panel be administered upon ICU admission and steroids be given at the discretion of the primary clinicians. We propose that the information provided by multiple IP-10 measurements performed routinely within the clinical setting, as opposed to a one-time cytokine panel, serves as actionable and dynamic support to immunomodulatory treatment decision-making by the primary team. Of note, since this manuscript was written, all protocols for managing COVID-19 patients have been updated several times to align with emerging evidence and expanded understanding of the disease.

The non-specific inflammatory biomarkers CRP and ferritin are used to determine the inflammatory status of COVID-19 patients. IP-10 represents a complementary tool that has the added advantage of rapidly indicating response to even a single dose of corticosteroid therapy, enabling its use as an aid to personalized corticosteroid treatment regimens. In addition, unlike CRP and ferritin, IP-10 correlated well with mortality among ICU patients, likely reflecting its purported role as an effector of acute lung injury in COVID-19 disease progression. Of note, this study did not include many COVID-19 patients at the early stages of disease progression. We hypothesize that serial measurement of IP-10 levels in such patients may flag those who are transitioning to the hyperinflamed state and enable early initiation of corticosteroid treatment in this subset of SARS-CoV-2-positive patients. Further studies are warranted to address this premise directly and to determine the optimal threshold of IP-10 levels that serves as an objective indicator of hyperinflammation. Another marker used to monitor inflammation is IL-6, which is the therapeutic target of tocilizumab, but its use as an indicator of inflammatory status may be constrained when the patients are under tocilizumab therapy [39]. In line with previous reports, we did not find that IL-6 expression was a predictor of mortality [26].

We also found that TRAIL levels may flag the inability to clear the virus and accordingly, correlate with disease severity. These data align with those of our previous study [36] and with preliminary data from an expanded multi-cohort analysis that is ongoing. Like IP-10, TRAIL can be measured rapidly and easily in one test run using a device recently CE marked for routine use for a related indication.

A recent meta-analysis of 24 observational studies from Asia, Europe, and North America encompassing 10,150 COVID-19 patients demonstrated a combined in-ICU mortality of 41.6% (95% confidence interval: 34.0–49.7%) [40]. We did not observe non-pulmonary organ failure and the in-ICU mortality rate was one out of 12. The main limitation of our study is that it was not a randomized controlled trial, thus the ability to estimate the magnitude of improvement in patient outcomes that is attributable to implementation of the dynamic clinical decision support protocol is limited. Moreover, the cohort study design does not allow evaluation of the contribution of various elements of the protocol, in particular the personalized corticosteroid regimens. Furthermore, this was a single-center study, constraining generalizability; additional studies of the utility of IP-10 for titrating corticosteroid treatments are warranted. The pivotal strength of our study is that it is the first prospective investigation of the potential value of serial IP-10 and TRAIL measurements as indicators of inflammatory status and viral clearance, respectively, in COVID-19 patients.

## Conclusions

Identification and management of severe COVID-19 patients presents a challenge to the treating physicians. Our study highlights the potential of serially measuring the levels of IP-10 to support objective decisions on immunomodulation of hyperinflamed patients within a multiparameter clinical decision support protocol. More work is required to establish the contribution of IP-10 to improved COVID-19 patient outcomes.

## Supporting information

S1 Fig The number of days CRP levels exceeding 90 mg/L is not correlated with mortality in patients admitted to the intensive care unit (n = 12; upper panel). The number of days of ferritin levels exceeding 500 ng/dL is not correlated with mortality in patients admitted to the intensive care unit (n = 12; lower panel). CRP and ferritin were measured at multiple time points during ICU stays. At least one CRP measurement exceeding 90 mg/L or ferritin measurement exceeding 500 ng/dL on a given day was sufficient for the analysis. Each circle represents a patient.
(TIF)

S2 Fig CRP (upper panel) and ferritin (lower panel) levels after initiation of corticosteroid therapy in ICU patients, n = 12. CRP and ferritin were measured at the indicated time points during ICU stays. Day 0 indicates initiation of corticosteroid therapy. Of note, at study initiation, some patients were studied who were already hospitalized in the wards and ICU. Corticosteroid treatment was not initiated prior to hospitalization. From the point of patient selection, every measurement taken is included in the graph.
(TIF)

S3 Fig The number of days of interleukin 6 (IL-6) levels exceeding 80 pg/mL is not correlated with mortality in patients admitted to the intensive care unit (n = 12; upper panel). The number of days of IL-6 levels exceeding 100 pg/mL is not correlated with mortality in patients admitted to the intensive care unit (n = 12; lower panel). IL-6 was measured at multiple time points during ICU stays. At least one IL-6 measurement exceeding 80 or 100 pg/mL on a given day was sufficient for the analysis. Each circle represents a patient.
(TIF)

S1 Table Characteristics of COVID-19 patients stratified by serum levels of IP-10, (total n = 52), IP-10 < 1,000 pg/ml (n = 33) and IP-10 > 1,000 pg/ml (n = 19). BMI, Body mass index; AST, Aspartate transaminase; AL, Alanine transaminase; LDH, Lactate dehydrogenase; Lymph. Abs, Absolute lymphocytes; Neu. Abs, Absolute neutrophils; PLT, platelets; PaO2/FiO2, Ratio of arterial oxygen partial pressure to fractional inspired oxygen; ECMO, Extracorporeal membrane oxygenation; qSOFA, quick sequential organ failure assessment; TRAIL, TNF-related apoptosis inducing ligand; IP-10, interferon-γ induced protein 10 (also known as CXCL-10); CRP, C-reactive protein.
(DOCX)
