# Supplementary figures and images for: Observational cohort study of IP-10’s potential as a biomarker to aid in inflammation regulation within a clinical decision support protocol for patients with severe COVID-19
Source: PLoS One. 2021 Jan 12;16(1):e0245296. doi: 10.1371/journal.pone.0245296 (PMC7802954; doi:10.1371/journal.pone.0245296)

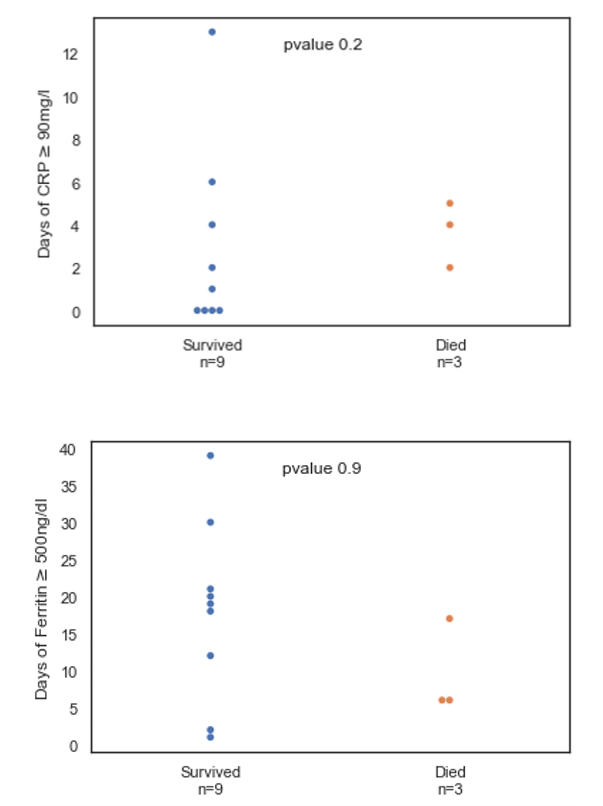

Supplement: S1 Fig — The number of days of ferritin levels exceeding 500 ng/dL is not correlated with mortality in patients admitted to the intensive care unit (n = 12; lower panel). CRP and ferritin were measured at multiple time points during ICU stays. At least one CRP measurement exceeding 90 mg/L or ferritin measurement exceeding 500 ng/dL on a given day was sufficient for the analysis. Each circle represents a patient. (TIF) [file pone.0245296.s001.tif]

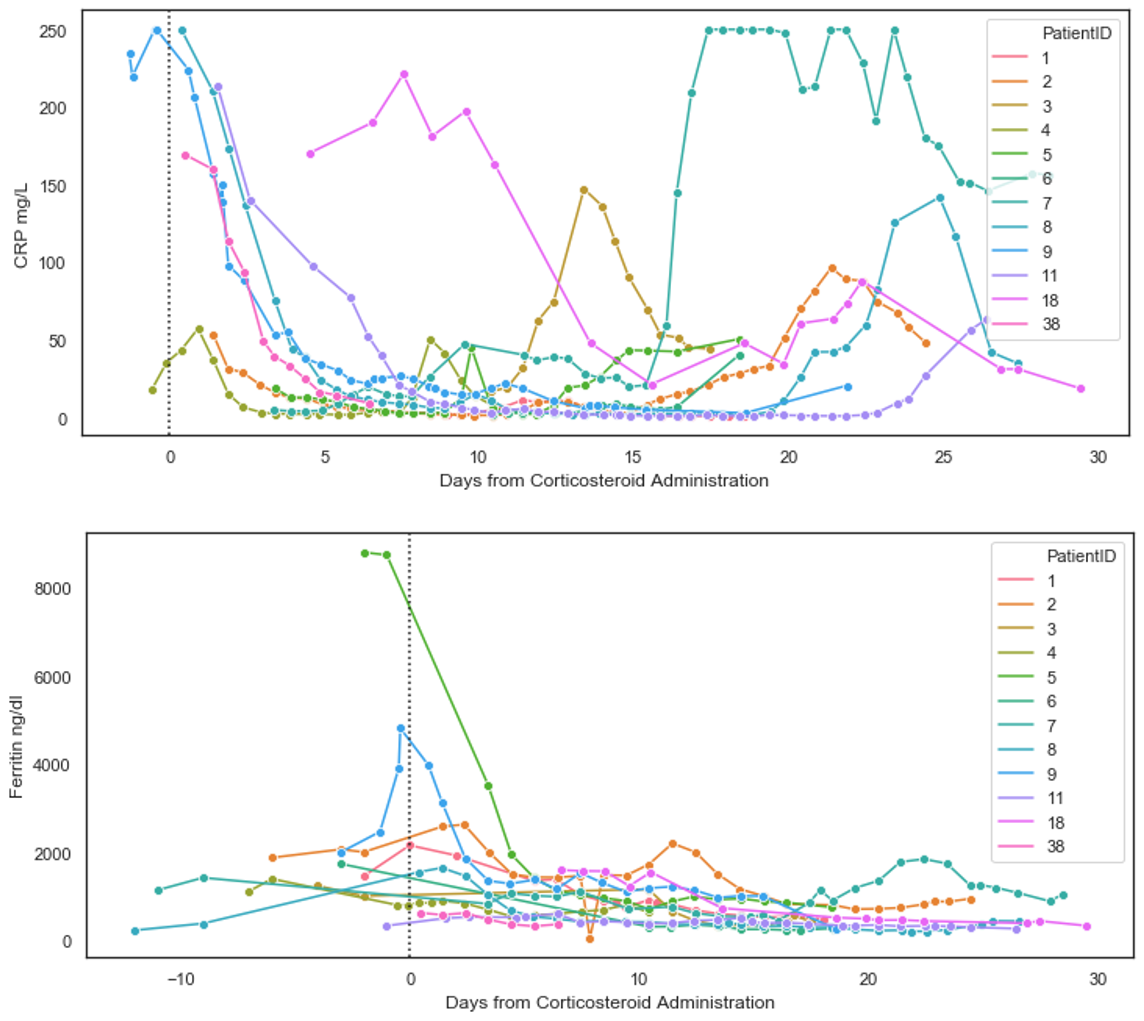

Supplement: S2 Fig — CRP and ferritin were measured at the indicated time points during ICU stays. Day 0 indicates initiation of corticosteroid therapy. Of note, at study initiation, some patients were studied who were already hospitalized in the wards and ICU. Corticosteroid treatment was not initiated prior to hospitalization. From the point of patient selection, every measurement taken is included in the graph. (TIF) [file pone.0245296.s002.tif]

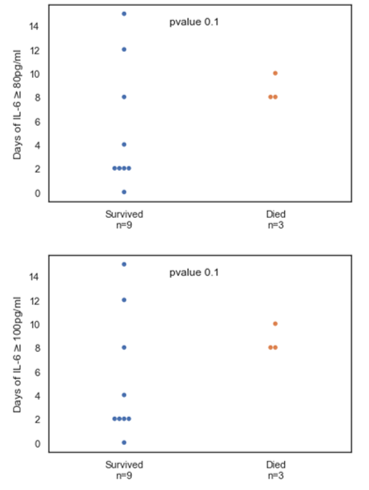

Supplement: S3 Fig — The number of days of IL-6 levels exceeding 100 pg/mL is not correlated with mortality in patients admitted to the intensive care unit (n = 12; lower panel). IL-6 was measured at multiple time points during ICU stays. At least one IL-6 measurement exceeding 80 or 100 pg/mL on a given day was sufficient for the analysis. Each circle represents a patient. (TIF) [file pone.0245296.s003.tif]
