# Supplementary material for: Observational cohort study of IP-10’s potential as a biomarker to aid in inflammation regulation within a clinical decision support protocol for patients with severe COVID-19
Source: PLoS One. 2021 Jan 12;16(1):e0245296. doi: 10.1371/journal.pone.0245296 (PMC7802954; doi:10.1371/journal.pone.0245296)
Supplement: S1 Table — BMI, Body mass index; AST, Aspartate transaminase; AL, Alanine transaminase; LDH, Lactate dehydrogenase; Lymph. Abs, Absolute lymphocytes; Neu. Abs, Absolute neutrophils; PLT, platelets; PaO2/FiO2, Ratio of arterial oxygen partial pressure to fractional inspired oxygen; ECMO, Extracorporeal membrane oxygenation; qSOFA, quick sequential organ failure assessment; TRAIL, TNF-related apoptosis inducing ligand; IP-10, interferon-γ induced protein 10 (also known as CXCL-10); CRP, C-reactive protein. (DOCX) [file pone.0245296.s004.docx]

**S1 Table:** Baseline characteristics of COVID-19 patients (total n = 52), IP-10 < 1,000 pg/ml (n = 33) and IP-10 > 1,000 pg/ml (n = 19)

|  | **0<IP-10<=1000 (n=33)** | **IP-10>1000 (n=19)** | **p-value** |
| --- | --- | --- | --- |
| **General** | | | |
| Age | 68.8 (21.5) | 68.5 (14.2) | 0.388 |
| Female | 12 (36.0%) | 4 (21.0%) | 0.353 |
| **Smoking Status** | | | |
| Smoker | 1 (3.0%) | 2 (11.0%) | 0.546 |
| Past Smoker | 2 (6.0%) | 1 (5.0%) | 1 |
| **Other** | | | |
| Death | 2 (6.0%) | 2 (11.0%) | 0.617 |
| Discharged | 32 (97.0%) | 19 (100.0%) | 1 |
| Highest Temp | 37.2 (1.0) | 38.1 (1.3) | 0.001 |
| Duration of Hospital Stay | 9.0 (13.8) | 12.0 (15.0) | 0.052 |
| Time between PCR Positive and BV Test (days) | 5.0 (6.0) | 3.0 (5.0) | 0.051 |
| ICU | 4 (12.0%) | 8 (42.0%) | 0.019 |
| Ventilated | 6 (18.0%) | 6 (32.0%) | 0.317 |
| Time between Symptoms and BV Test (days) | 7.0 (12.0) | 7.0 (8.0) | 0.199 |
| Time between Symptoms and ICU Admission (days) | 8.0 (2.8) | 8.5 (5.5) | 0.244 |
| Time between Symptoms and PCR Positive (days) | 2.0 (3.0) | 4.0 (4.5) | 0.255 |
| Time on Ventilation (days) | 0.0 (0.0) | 0.0 (12.5) | 0.174 |
| Time between PCR Positive and ICU Admission (days) | 4.0 (2.2) | 4.5 (1.8) | 0.265 |
| Time between PCR Positive and PCR Negative (days) | 18.0 (11.8) | 25.0 (13.2) | 0.112 |
| **Co-infection** | | | |
| Bacterial Infection | 4 (12.0%) | 6 (32.0%) | 0.142 |
| Co-infection | 4 (12.0%) | 6 (32.0%) | 0.142 |
| Fungal Infection | 2 (6.0%) | 2 (11.0%) | 0.617 |
| **Conditions** | | | |
| Diabetes | 13 (39.0%) | 9 (47.0%) | 0.771 |
| Cardiovascular Disease | 7 (21.0%) | 2 (11.0%) | 0.458 |
| Cerebrovascular disease | 3 (9.0%) | 0 (0.0%) | 0.291 |
| Chronic Lung Disease | 3 (9.0%) | 4 (21.0%) | 0.4 |
| Chronic renal disease | 4 (12.0%) | 2 (11.0%) | 1 |
| Dyslipidemia | 4 (12.0%) | 5 (26.0%) | 0.26 |
| Hypertension | 11 (33.0%) | 13 (68.0%) | 0.021 |
| Hypothyroidism | 2 (6.0%) | 4 (21.0%) | 0.175 |
| Malignancy | 1 (3.0%) | 4 (21.0%) | 0.054 |
| Obesity (BMI>30 kg/m2) | 7 (21.0%) | 2 (11.0%) | 0.458 |
| Immunodeficiency | 1 (3.0%) | 0 (0.0%) | 1 |
| **Symptoms** | | | |
| Sore throat | 1 (3.0%) | 0 (0.0%) | 1 |
| Vomiting | 0 (0.0%) | 2 (11.0%) | 0.129 |
| Urinary Complaints | 1 (3.0%) | 0 (0.0%) | 1 |
| Sputum production | 1 (3.0%) | 0 (0.0%) | 1 |
| Weakness | 8 (24.0%) | 4 (21.0%) | 1 |
| Rhinorrhea | 1 (3.0%) | 0 (0.0%) | 1 |
| Lethargy | 2 (6.0%) | 0 (0.0%) | 0.527 |
| Agitation | 0 (0.0%) | 1 (5.0%) | 0.365 |
| Chest pain | 2 (6.0%) | 0 (0.0%) | 0.527 |
| Confusion | 2 (6.0%) | 0 (0.0%) | 0.527 |
| Chills | 1 (3.0%) | 0 (0.0%) | 1 |
| Diarrhea | 4 (12.0%) | 2 (11.0%) | 1 |
| Dyspnea | 12 (36.0%) | 11 (58.0%) | 0.157 |
| Fatigue | 1 (3.0%) | 2 (11.0%) | 0.546 |
| Fever | 16 (48.0%) | 13 (68.0%) | 0.247 |
| Headache | 4 (12.0%) | 1 (5.0%) | 0.641 |
| Loss of Taste and Smell | 4 (12.0%) | 1 (5.0%) | 0.641 |
| Myalgia | 4 (12.0%) | 0 (0.0%) | 0.284 |
| Nausea | 2 (6.0%) | 1 (5.0%) | 1 |
| Cough | 15 (45.0%) | 11 (58.0%) | 0.565 |
| **Markers** | | | |
| AST (GOT) (u/l)_max | 26.6 (35.2) | 55.7 (37.9) | 0.005 |
| ALT (GPT) (u/l)_max | 28.6 (46.1) | 63.4 (85.1) | 0.017 |
| Albumin (g/dl)_max | 3.8 (0.6) | 3.7 (0.3) | 0.082 |
| INR_max | 1.1 (0.2) | 1.1 (0.3) | 0.343 |
| Glucose (mg/dl)_max | 131.2 (110.8) | 184.4 (150.3) | 0.017 |
| LDH (u/l)_max | 491.0 (312.8) | 689.0 (266.0) | 0.011 |
| Lymph.abs (K/micl)_max | 1.3 (0.8) | 1.3 (0.9) | 0.402 |
| Lymph.abs (K/micl)_min | 0.9 (0.7) | 0.5 (0.4) | 0.004 |
| Neu.abs (K/micl)_max | 5.1 (3.8) | 8.0 (8.5) | 0.014 |
| Neu.abs (K/micl)_min | 3.7 (2.5) | 2.8 (1.5) | 0.045 |
| PLT (10^3)_max | 374.0 (202.0) | 375.0 (158.5) | 0.421 |
| PLT (10^3)_min | 229.0 (129.0) | 162.0 (55.0) | 0.013 |
| PaO2/FiO2_max | 238.0 (84.0) | 344.5 (136.5) | 0.388 |
| Total protein (g/dl)_max | 7.0 (0.7) | 7.0 (0.7) | 0.406 |
| PaO2/FiO2_min | 83.0 (63.0) | 89.5 (46.5) | 0.44 |
| Troponin_max | 12.0 (33.0) | 25.0 (40.0) | 0.059 |
| Urea_max | 34.6 (38.0) | 58.0 (57.9) | 0.002 |
| Ferritin_min | 281.1 (338.8) | 313.3 (365.0) | 0.202 |
| Ferritin_max | 449.4 (697.1) | 901.0 (1147.6) | 0.005 |
| D-Dimer_max | 1173.5 (2575.2) | 2175.0 (6061.5) | 0.055 |
| Creatinine (mg/dl)_max | 0.9 (0.4) | 1.0 (0.4) | 0.008 |
| Bili total (mg/dl)_max | 0.4 (0.4) | 0.8 (0.6) | 0.018 |
| CRP_min (mg/l) | 20.3 (54.2) | 19.0 (52.2) | 0.458 |
| Median CRP (mg/l) | 38.4 (70.4) | 107.4 (88.8) | 0 |
| CRP_max (mg/l) | 66.1 (82.3) | 169.5 (109.0) | 0 |
| **Scores** | | | |
| COVID-19 Severity | 11 (33.0%) | 15 (79.0%) | 0.003 |
| qSOFA (on admission) | 0.0 (1.0) | 1.0 (1.0) | 0.064 |
| **Treatments** | | | |
| Solumedrol | 4 (12.0%) | 8 (42.0%) | 0.019 |
| Hydrocortisone | 5 (15.0%) | 5 (26.0%) | 0.467 |
| Systemic Steroids (IV/PO) | 10 (30.0%) | 12 (63.0%) | 0.04 |
| Renal Replacement Therapy | 1 (3.0%) | 1 (5.0%) | 1 |
| Remdesivir | 0 (0.0%) | 1 (5.0%) | 0.365 |
| Prednisone | 3 (9.0%) | 3 (16.0%) | 0.656 |
| Nitric Oxide | 2 (6.0%) | 3 (16.0%) | 0.342 |
| ECMO | 1 (3.0%) | 0 (0.0%) | 1 |
| Vasopressors | 5 (15.0%) | 6 (32.0%) | 0.181 |
| Convalescent Plasma | 1 (3.0%) | 6 (32.0%) | 0.007 |
| Azithromycin | 16 (48.0%) | 19 (100.0%) | >0.001 |
| Antiviral Lopinavir/Ritonavir | 0 (0.0%) | 7 (37.0%) | >0.001 |
| Antiviral Hydroxychloroquine plus Azithromycin | 14 (42.0%) | 19 (100.0%) | >0.001 |
| Antiviral Hydroxychloroquine | 17 (52.0%) | 19 (100.0%) | >0.001 |
| Antibiotics | 15 (45.0%) | 15 (79.0%) | 0.023 |
| Tocilizumab | 5 (15.0%) | 8 (42.0%) | 0.047 |
| Days under Systemic Steroids | 0.0 (3.0) | 5.0 (10.5) | 0.007 |
| **First BV test** | | | |
| TRAIL (pg/ml) | 58.2 (22.5) | 50.7 (52.7) | 0.131 |
| CRP (mg/l) | 38.4 (70.4) | 107.4 (88.8) | >0.001 |
| IP-10 (pg/ml) | 333.2 (419.5) | 1984.5 (1525.6) | >0.001 |

**S1 Table**: BMI, Body mass index; AST, Aspartate transaminase; AL, Alanine transaminase; LDH, Lactate dehydrogenase; Lymph. Abs, Absolute lymphocytes; Neu. Abs, Absolute neutrophils; PLT, platelets; PaO2/FiO2, Ratio of arterial oxygen partial pressure to fractional inspired oxygen; ECMO, Extracorporeal membrane oxygenation; qSOFA, quick sequential organ failure assessment; TRAIL, TNF-related apoptosis inducing ligand; IP-10, interferon-γ induced protein 10 (also known as CXCL-10); CRP, C-reactive protein.
